# Supplementary material for: Standardized Digital Image Analysis of PD-L1 Expression in Head and Neck Squamous Cell Carcinoma Reveals Intra- and Inter-Sample Heterogeneity with Therapeutic Implications
Source: Cancers (Basel). 2024 May 31;16(11):2103. doi: 10.3390/cancers16112103 (PMC11171694; doi:10.3390/cancers16112103)
Supplement: Supplementary file 1 [file cancers-16-02103-s001.zip › cancers-3022546-supplementary.pdf]

Standardized Digital Image Analysis of PD-L1 Expression in Head and Neck Squamous Cell Carcinoma Reveals Intra- and Inter-Sample Heterogeneity with Therapeutic Implications

Eric Deuss, Cornelius Kürten, Lara Fehr, Laura Kahl, Stefanie Zimmer, Julian Künzel, Roland H. Stauber, Stephan Lang, Timon Hussain and Sven Brandau

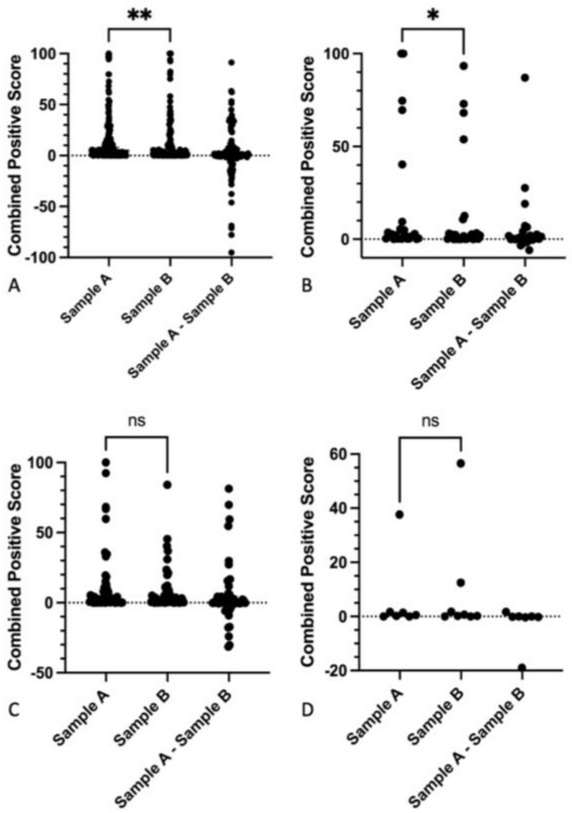

Figure S1. Intratumoral heterogeneity of CPS and TPS between two samples from the same specimen of primary tumors and local recurrences (**A-D**) Graphs show single plotted values of CPS. (**A**) Primary tumors and (**B**) local recurrent tumors showed significant differences of combined positive score (** $p < 0.01$; * $p \leq 0.05$); (**C**) lymph node metastasis and (**D**) distant metastasis offered no differences of CPS (ns $p > 0.05$).

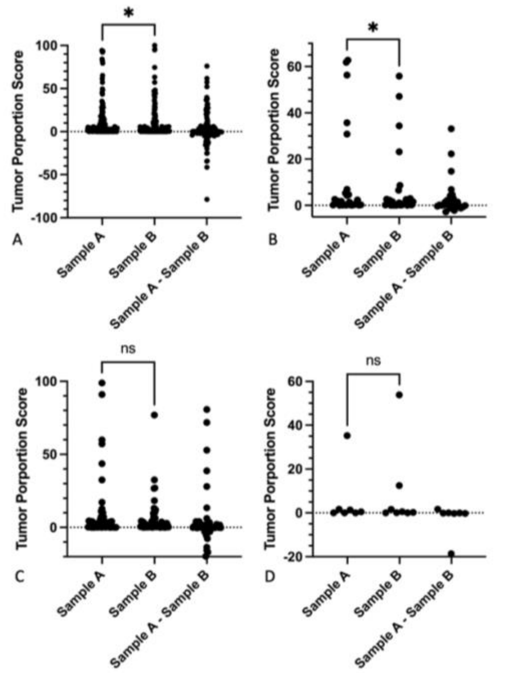

Figure S2. Intratumoral heterogeneity of CPS and TPS between two samples from the same specimen of lymph node and distant metastasis (**A-D**) Graphs show single plotted values of TPS. (**A**) Primary tumors and (**B**) local recurrent tumors showed significant differences of TPS (* $p \leq 0.05$); (**C**) lymph node metastasis and (**D**) distant metastasis offered no differences of CPS (ns $p > 0.05$).

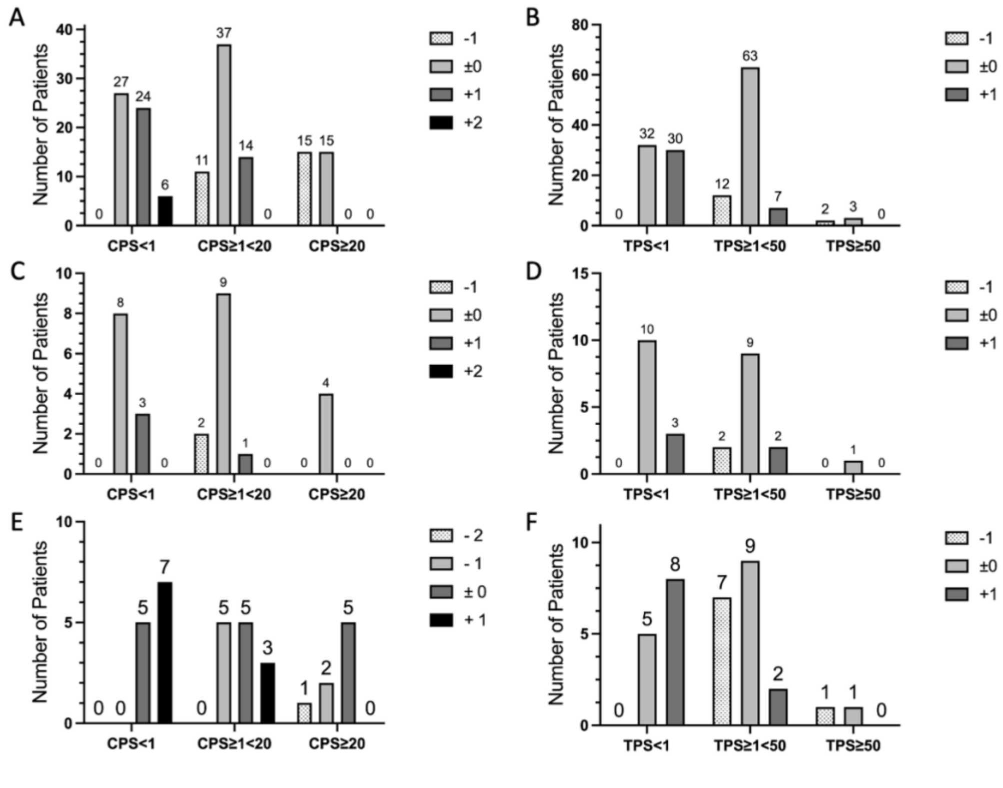

Figure S3. Description of intratumoral heterogeneity by absolute number of changes between treatment relevant classes of CPS and TPS in primary tumors, local recurrent tumors and lymph node metastases (**A,C,E**) Graphs show changes (-2; -1; ±0; +1; +2) of

combined positive score (CPS) categories (< 1; ≥ 1 < 20; ≥ 20) between two different intratumoral biopsies of (**A**) primary tumors, (**C**) local recurrent tumors and (**E**) lymph node metastasis.; (**B,D,F**) Graphs show changes (-1; ±0; +1) of tumor proportion score (TPS) categories (< 1 %; ≥ 1 % < 50 %; ≥ 50 %) between two different intratumoral biopsies of (**B**) primary tumors, (**D**) local recurrent tumors and (**F**) lymph node metastasis.

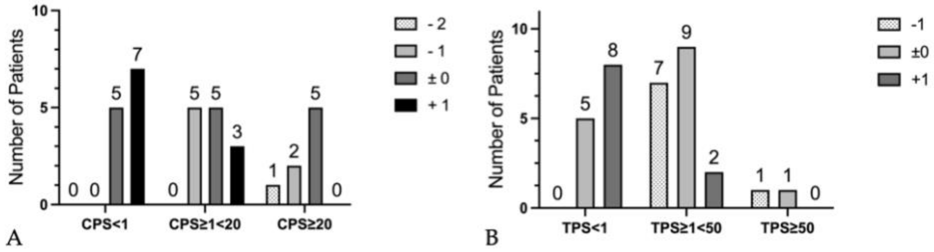

Figure S4. Description of intertumoral heterogeneity by absolute number of changes between treatment relevant classes of CPS and TPS between primary tumors and lymph node metastases (**A**,**B**) Graphs show frequencies of tricategorical changes in (**A**) CPS and (**B**) TPS among primary tumors and lymph node metastasis.

Table S1. Analyzing parameter for positive cell detection in QuPath v3.4 .

**Setup parameter**	
Detection image	Hematoxylin OD
Requested pixel size	0.5 µm
**Nucleus parameter**	
Background radius	8 µm
Median filter raidus	0 µm
Sigma	1,5 µm
Minum area	10 µm^2
Maximum area	400 µm^2
**Intensity parameters**	
Thershold	0.1
Max background intensit	2
Split by shape	On
Exclude DAB	Off
**Cell parameters**	
CellExpension	10 µm
Include cell nucelus	On
**General parameters**	
Smooth boundaries	On
Make measurements	On
**Intensity threshold parameters**	
Score compartment	Cell: DAB OD mean
Threshold 1+	0.15
Single Threshold	On

Table S2. Results of non-parametric spearman correlation for CPS and TPS among different tumor kinds.

	N	rho	p-value	95 % confidence intervall
CPS primary tumor vs. CPS local recurrent tumor	12	-0.21	0.51	-0.76 - 0.49
CPS primary tumor vs. CPS lymphnode metastasis	32	0.47	0.01	0.14 - 0.71
CPS primary tumor vs. CPS distant metastasis	4	1.00	0.03	-
CPS lymphnode metastasis vs. CPS local recurrent tumor	6	0.31	0.54	-0.69 - 0.90
TPS primary tumor vs. TPS local recurrent tumor	12	-0.14	0.31	-0.72 - 0.55
TPS primary tumor vs. TPS lymphnode metastasis	32	0.50	0.01	0.15 - 0.74
TPS primary tumor vs. TPS distant metastasis	4	1.00	0.00	-
TPS lymphnode metastasis vs. TPS local recurrent tumor	6	0.09	0.87	-0.79 - 0.85
CPS primary tumor vs. TPS primary tumor	159	0.98	0.00	0.97 - 0.98
CPS lymphnode metastasis vs. TPS lymphnode metastasis	32	0.99	0.00	0.97 - 0.99
CPS local recurrent tumor vs. TPS local recurrent tumor	12	0.85	0.00	0.53 - 0.96
CPS distant metastasis vs. TPS distant metastasis	4	1.00	0.00	-

Table S3. Results of ordinary correlation between tricategorical CPS or TPS (< 1 vs. ≥ 1) and clinicopathological features.

		**Tricategorial CPS**		**Tricategorial TPS**	
**Parameter**	N	Kendall Tau B	p value	Kendall Tau B	p value
**Age** (< 60 vs. ≥ 60)	161	-0.02	0.80	0.04	0.57
**T category** (1/2 vs. 3/4)	163	-0.15	0.04	-0.17	0.03
**N category** (negative vs. positive)	163	-0.09	0.23	-0.14	0.05
**UICC stage 7th edition** (I-II vs. II-IV)	164	-0.06	0.40	-0.15	0,03
**Lymphnoode Ratio** (< 0.1 vs. > 0.1)	88	0.14	0.31	0.07	0.32
**Extranodal extension** (negative vs. positive)	42	-0.20	0.18	-0.15	0.29
**Smoking history** (negative vs. positive)	106	0.06	0.55	-0.04	0.67
**Alcohol abuse** (negative vs. positive)	106	-0.10	0.26	-0.05	0.90
**p16 status** (negative vs. positive)	100	0.12	0.21	-0.00	0.98
**Grading** (I vs. II/III)	101	0.14	0.17	0.03	0.75

Table S4. Results of dichotomous correlation between bicategorical CPS or TPS (< 1 vs. ≥ 1) and clinicopathological features.

		**Bicategorial CPS**		**Bicategorial TPS**	
**Parameter**	N	Phi	p value	Phi	p value
**Sex** (female vs. Male)	168	-0.125	0.11	-0.06	0.44
**Age** (< 60 vs. ≥ 60)	161	0.04	0.65	0.00	0.96
**T category** (1/2 vs. 3/4)	161	-0.18	0.02	-0.17	0.03
**N category** (negative vs. positive)	163	-0.16	0.04	-0.19	0.02
**UICC stage 7th edition** (I-II vs. II-IV)	164	-0.14	0.07	-0.18	0.02
**Lymphnoode Ratio** (< 0.1 vs. > 0.1)	88	0.07	0.53	0.08	0.43
**Extranodal extension** (negative vs. positive)	42	-0.14	0.36	-0.20	0.20
**Smoking history** (negative vs. positive)	106	0.06	0.51	-0.04	0.71
**Alcohol abuse** (negative vs. positive)	106	-0.10	0.29	-0.04	0.68
**p16 status** (negative vs. positive)	100	0.03	0.79	-0.04	0.73
**Grading** (I vs. II/III)	101	0.04	0.53	-0.03	0.78
